# Successful management of severe anaerobic infection inducing mediastinal and subcutaneous emphysema, septic shock, and ARDS: a case report highlighting VV-ECMO and NGS-guided therapy

**DOI:** 10.3389/fmed.2025.1568410

**Published:** 2025-03-17

**Authors:** Haohao Wu, Pin Lan, Kechun Zhou, Xingzhen Wu, Lutao Xie

**Affiliations:** Department of Emergency, Lishui Central Hospital, The Fifth Affiliated Hospital of Wenzhou Medical University, Lishui, China

**Keywords:** anaerobic infection, mediastinal emphysema, subcutaneous emphysema, septic shock, acute respiratory distress syndrome (ARDS), VV-ECMO, airway management, NGS (next-generation sequencing)

## Abstract

**Background:**

Mediastinal and cervical subcutaneous emphysema caused by anaerobic infections is rare in clinical practice, particularly when accompanied by sepsis, septic shock, and severe acute respiratory distress syndrome (ARDS). These cases pose significant treatment challenges. Veno-venous extracorporeal membrane oxygenation (VV-ECMO), as a life-saving intervention, has been increasingly utilized in patients with severe infections and refractory hypoxemia. This report aims to evaluate the effectiveness of VV-ECMO in the treatment of mediastinal and subcutaneous emphysema, sepsis, and severe ARDS caused by anaerobic infections, and to summarize relevant therapeutic strategies.

**Case presentation:**

A 49-year-old male was admitted with fever, sore throat, chest tightness, and hoarseness. On admission, he presented with severe hypoxemia, sepsis, and acute kidney injury. Chest computed tomography (CT) revealed bilateral mediastinal emphysema and cervical subcutaneous emphysema. Next-generation sequencing (NGS) confirmed an anaerobic bacterial infection. Despite high-flow oxygen therapy and antibiotic treatment, the patient’s oxygenation continued to deteriorate, culminating in cardiopulmonary arrest. VV-ECMO was initiated to improve oxygenation, alongside prone positioning ventilation, sputum clearance, and alveolar lavage. After 7 days of ECMO support and anti-infective treatment, the patient’s oxygenation improved significantly, inflammatory markers decreased, and ECMO was successfully weaned.

**Conclusion:**

VV-ECMO is of critical value in managing septic shock and ARDS caused by severe anaerobic infections, effectively improving oxygenation and supporting organ function. This case highlights the pivotal role of airway management, VV-ECMO support, and comprehensive therapeutic strategies in the management of complex infectious ARDS, providing valuable insights for similar clinical scenarios.

## Background

Mediastinal emphysema and cervical subcutaneous emphysema are rare in clinical practice, particularly when caused by anaerobic infections and accompanied by sepsis and severe acute respiratory distress syndrome (ARDS). Anaerobic bacteria release gases through anaerobic metabolism in hypoxic environments, leading to further tissue damage and the spread of infection (1). These infections typically progress rapidly, and without timely intervention, they can result in severe systemic complications.

In recent years, next-generation sequencing (NGS) technology has been widely applied to the detection of pathogens in infectious diseases, particularly in cases involving complex polymicrobial infections or where the sensitivity of conventional culture methods is limited (2). NGS allows for high-throughput genomic sequencing, enabling precise identification of pathogens and the detection of antimicrobial resistance genes, thereby providing critical guidance for personalized antimicrobial therapy. Additionally, veno-venous extracorporeal membrane oxygenation (VV-ECMO) serves as a vital support strategy for patients with severe ARDS, offering an effective means to improve oxygenation when conventional treatments fail (3). In this case, NGS was employed to identify the causative pathogens, and VV-ECMO was successfully utilized to treat the patient, offering valuable insights for the management of similar clinical scenarios.

## Case presentation

A 49-year-old male presented to the emergency department with complaints of fever and sore throat for 3 days, worsening with chest tightness for 1 day. Three days prior, the patient experienced fever (maximum temperature 39°C), sore throat, and fatigue without any apparent cause, and he had not received treatment. One day before admission, his symptoms worsened, accompanied by chest tightness, neck pain, and hoarseness. At an outside hospital, he was diagnosed with “sepsis, acute renal failure, and mediastinal emphysema” and was transferred to our emergency intensive care unit (EICU) due to the severity of his condition.

**Figure 1:**
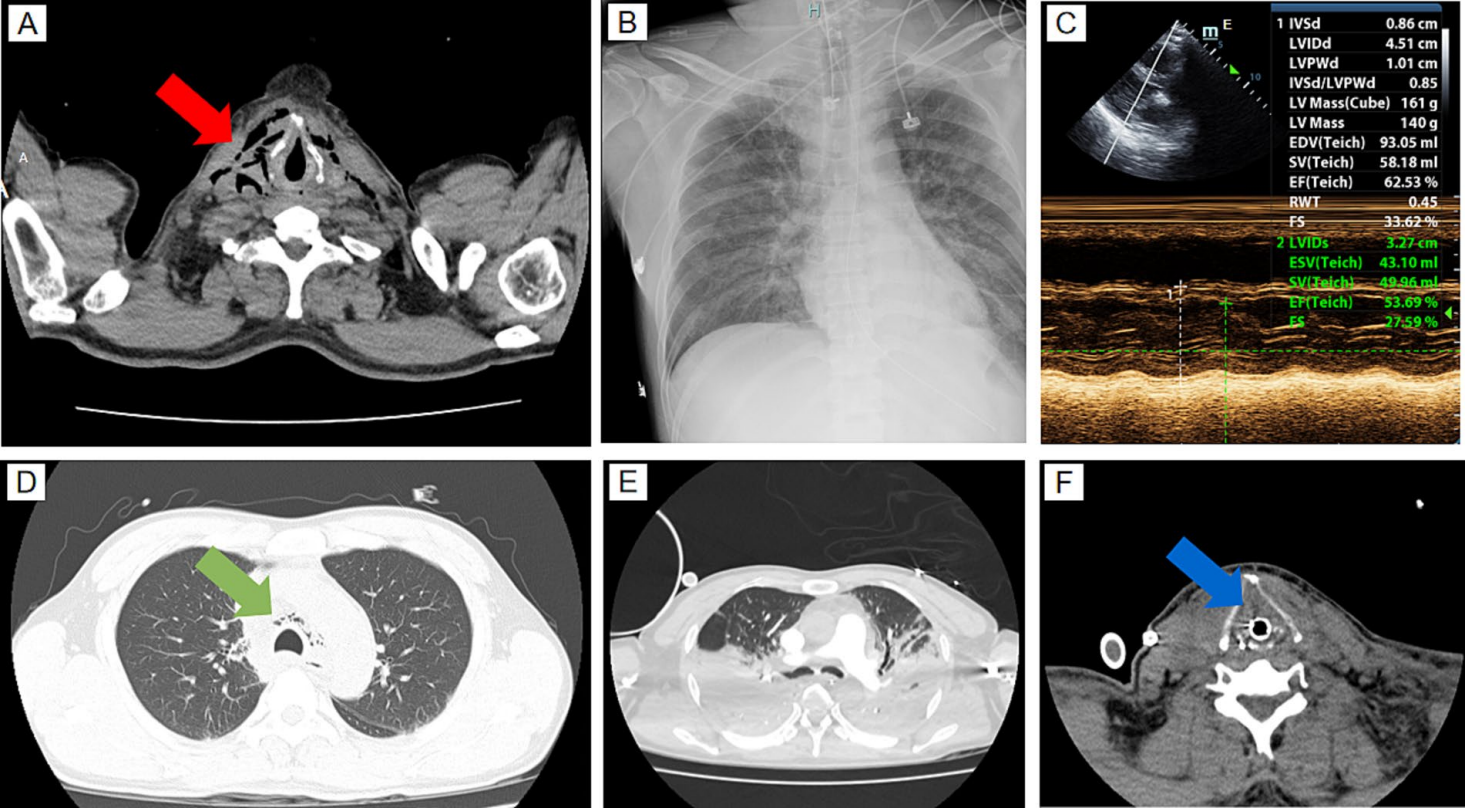
Imaging studies at admission and prior to ECMO initiation. **(A)** Chest CT scan at admission showing subcutaneous emphysema in the neck region (red arrow). **(B)** Chest X-ray before ECMO initiation showing diffuse bilateral lung shadows, indicating severe lung involvement. **(C)** Echocardiographic assessment before ECMO initiation showing an ejection fraction (EF) of 62.53%. **(D)** Chest CT scan at admission demonstrating mediastinal emphysema (green arrow). **(E)** Contrast-enhanced chest CT after ECMO initiation revealing worsening lung infection with consolidation. **(F)** Oropharyngeal CT scan after ECMO initiation showing neck soft tissue swelling and compression of the airway surrounding tissues (blue arrow).

Upon arrival at the emergency room, the patient had a temperature of 38.6°C, heart rate of 138 beats per minute, respiratory rate of 32 breaths per minute, and blood pressure of 120/72 mmHg. His oxygen saturation was 85% under 3 L/min nasal cannula oxygen. Physical examination revealed subcutaneous emphysema in the neck and upper chest. Auscultation showed reduced breath sounds in both lungs. Laboratory tests indicated severe sepsis (PCT >100 ng/mL), acute kidney injury (creatinine 514 μmol/L), lactic acidosis (lactate 4.7 mmol/L), myocardial injury (troponin 0.491 ng/mL), and hypoxemia (PaO₂ 72.4 mmHg). Chest CT revealed bilateral cervical subcutaneous emphysema, mediastinal emphysema, mild pulmonary infiltrates, and small pleural effusions (Figures 1A,D). The initial diagnosis was “sepsis, acute kidney injury, mediastinal emphysema, and cervical subcutaneous emphysema.” Immediate treatment included imipenem-cilastatin sodium (1.0 g IV), fluid resuscitation, high-flow oxygen therapy, and supportive care. However, the patient’s hypoxemia worsened, prompting admission to the EICU.

At the EICU, the patient’s temperature was 38.0°C, heart rate 139 beats per minute, blood pressure 105/73 mmHg, and respiratory rate 28 breaths per minute under high-flow oxygen, maintaining an oxygen saturation of 95%. The patient received high-flow oxygen support, intensive care, cardiac monitoring, and continued imipenem-cilastatin sodium (1.0 g IV every 12 h). Supportive care included airway clearance and gastric protection. To identify the causative pathogen, NGS and microbiological examinations were performed immediately after admission.

On day 2 at 7:00 a.m., the patient’s oxygenation further deteriorated, with blood gas analysis showing PaO₂ at 41.9 mmHg. Emergency orotracheal intubation was performed to improve oxygenation. Severe glottic edema was observed during intubation, resulting in difficult airway management and cardiopulmonary arrest. Cardiopulmonary resuscitation was initiated, and spontaneous circulation was restored within 2 min after epinephrine administration. Orotracheal intubation was successfully completed under fiberoptic bronchoscopy guidance. Mechanical ventilation was initiated in AC/VC mode with settings: respiratory rate 15 breaths per minute, tidal volume 550 mL, PEEP 10 cmH₂O, and FiO₂ 100%. Norepinephrine (0.53 μg/kg/min) was administered to maintain hemodynamic stability. Vancomycin (0.5 g IV every 12 h) was added to the antimicrobial regimen.

By the evening of day 2, the patient still required pure oxygen to maintain oxygen saturation at 91%, with hemodynamic support relying on norepinephrine. Bedside chest radiographs showed worsening bilateral pulmonary infiltrates (Figure 1B), and echocardiography revealed mildly reduced cardiac function (Figure 1C). As conventional mechanical ventilation failed to improve oxygenation, VV-ECMO cannulation was initiated at 22:59 and successfully commenced by 23:15. Post-ECMO imaging, including pulmonary artery CTA and oropharyngeal CT, was conducted. Pulmonary artery CTA ruled out pulmonary embolism but revealed significant bilateral lung consolidation, infection, and pleural effusion (Figure 1E). Oropharyngeal CT showed swelling of the neck soft tissue (Figure 1F). Based on these findings, pulmonary embolism was excluded, and the final diagnosis was sepsis, septic shock, and severe ARDS. Treatment was adjusted to continue the current antimicrobial regimen, with protective ventilation and prone positioning to further improve oxygenation under VV-ECMO support.

**Figure 2:**
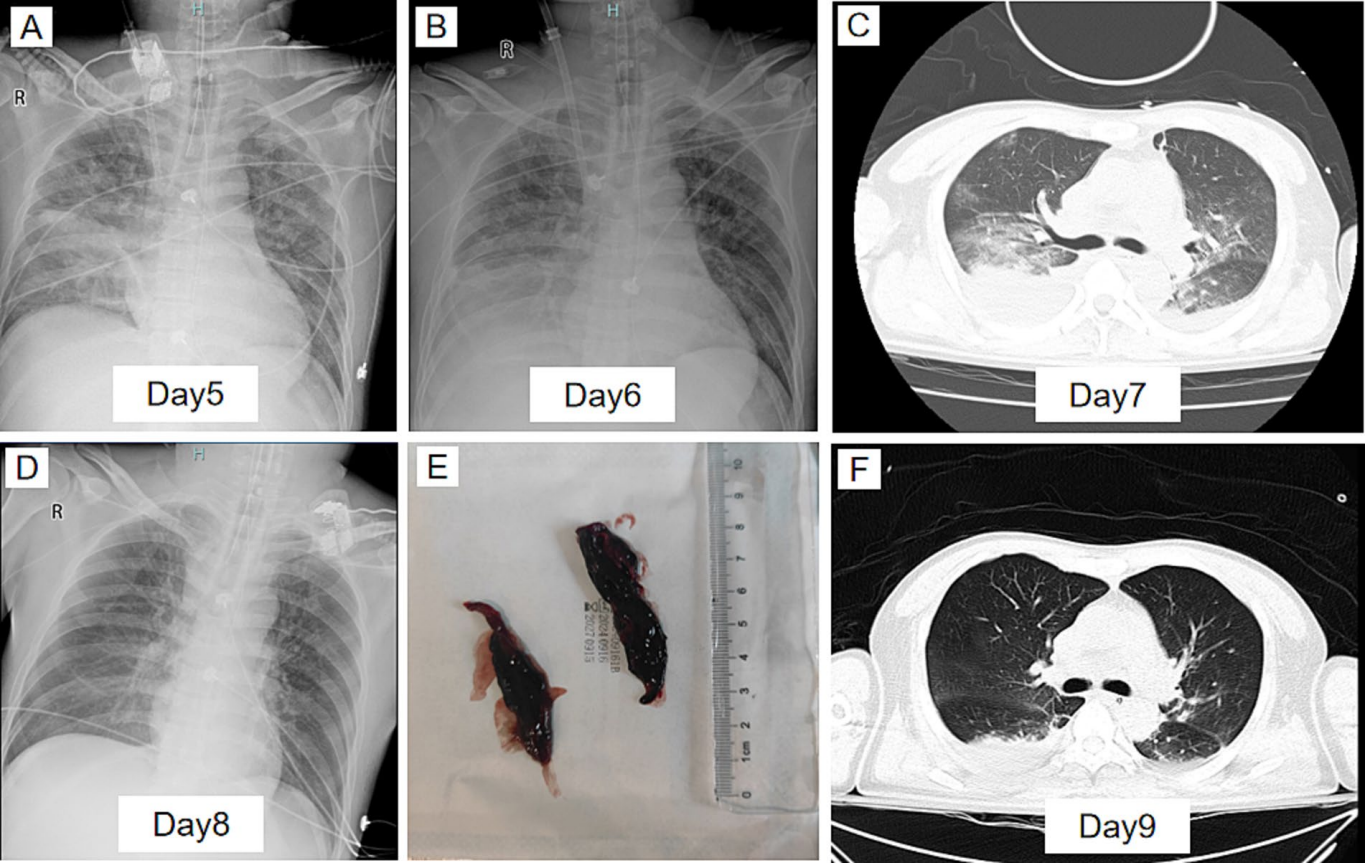
Dynamic imaging during and after VV-ECMO treatment. **(A)** Chest X-ray on day 5 showing progressive lung consolidation and bilateral infiltrates, indicating ongoing pulmonary infection. **(B)** Chest X-ray on day 6 showing persistent bilateral lung infiltrates with worsening pulmonary involvement. **(C)** Chest CT scan on day 7 showing significant consolidation in both lungs, indicating the progression of the pulmonary infection. **(D)** Chest X-ray on day 8 showing improvement in lung consolidation, with a reduction in pulmonary infiltrates. **(E)** Fiberoptic bronchoscopy showing blood clots aspirated from the airways. **(F)** Chest CT scan on day 9 after ECMO weaning showing significant improvement in lung conditions with reduced consolidation and improved airspaces.

**Figure 3:**
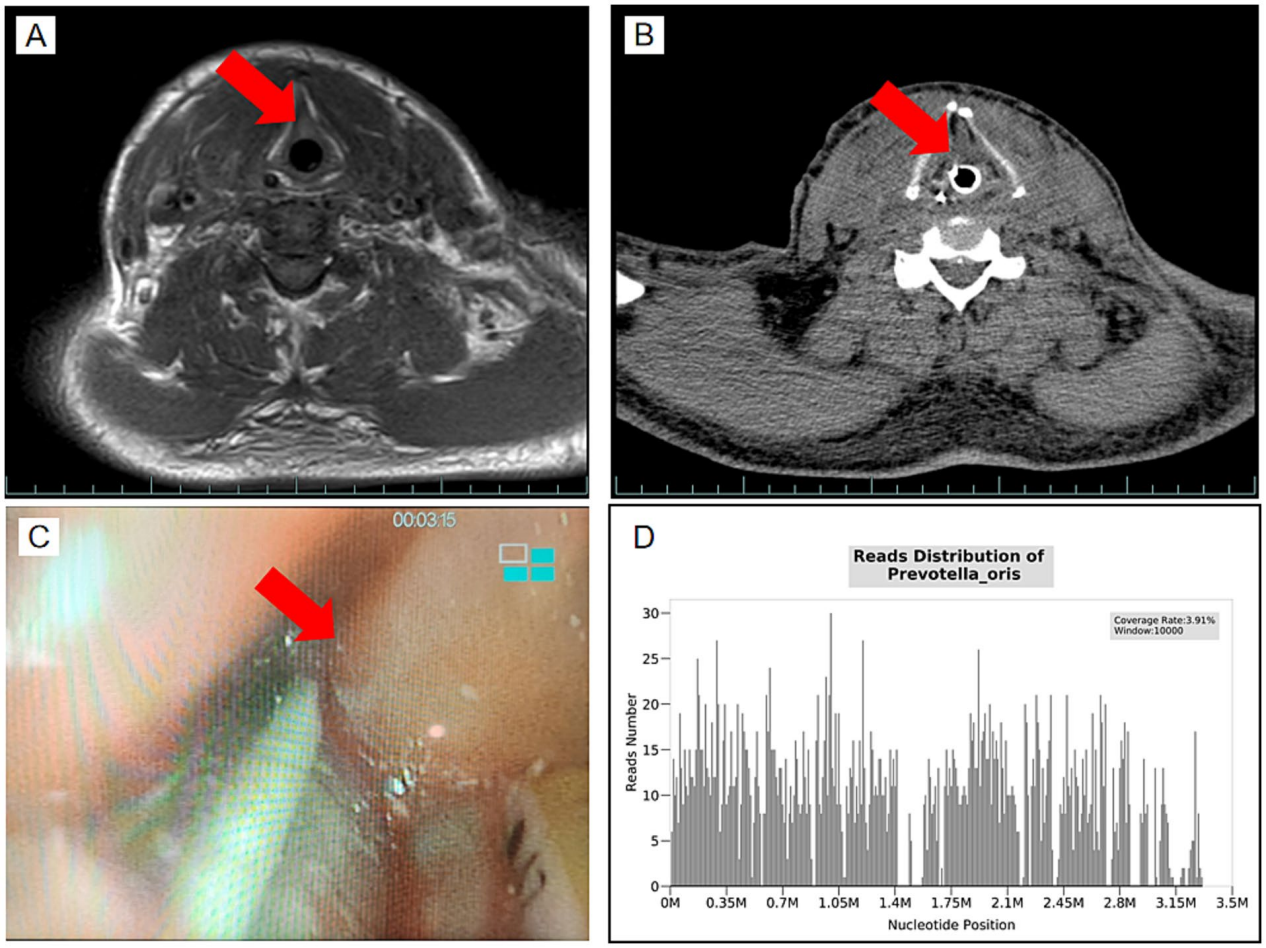
Neck swelling prior to extubation and NGS results. **(A)** Neck MRI scan showing mild soft tissue swelling in the cervical region (red arrow), indicating localized edema. **(B)** Neck CT scan showing soft tissue swelling and the presence of inflammation surrounding the airway (red arrow). **(C)** Laryngoscopy image showing significant edema of the vocal cords and surrounding tissue (red arrow), contributing to difficult extubation. **(D)** NGS results identifying *Prevotella oris*. *Prevotella oris* was detected by NGS with a coverage rate of 3.91%, indicating its presence among the anaerobic pathogens in the patient’s sample.

During VV-ECMO support, the blood flow was maintained at approximately 4.0 L/min, and gas flow was set at 4 L/min. Prone positioning ventilation was employed to optimize oxygenation and reduce pulmonary stress. Multiple fiberoptic bronchoscopies and alveolar lavages were performed, with large airway blood clots (up to 6 cm) successfully removed (Figure 2E). On day 4, NGS results identified anaerobic bacteria, primarily *Prevotella* species (Figure 3D), which are commonly associated with oropharyngeal infections. Their metabolic products were consistent with the mediastinal and subcutaneous emphysema observed. Based on these findings, the antimicrobial regimen of imipenem-cilastatin sodium and vancomycin was continued. Over the course of VV-ECMO treatment, inflammatory markers, including PCT and CRP, gradually declined, indicating improvement in the systemic inflammatory state (Table 1). Serial chest imaging showed a significant reduction in lung consolidation and gradual resolution of pulmonary infection (Figures 2A–D).

**Table 1:** Dynamic changes in hemodynamic, laboratory, ventilator, and ECMO parameters before ECMO initiation, during ECMO support, and after ECMO weaning.

	Pre-ECMO	ECMO day 1	ECMO day 3	ECMO day 6	Post-ECMO
Hemodynamic parameters					
Heart rate (bpm)	114	104	80	105	84
Arterial pressure (mmHg)	107	116	118	133	142
Norepinephrine (mcg/kg/min)	0.53	0.57	0.22	0	0
Laboratory inspection					
CRP (mg/L)	232	190	139	224	219
PCT (ng/mL)	>100	>100	75	9.2	3.5
pCO_2_ (mmHg)	43.4	28.3	37.3	34.7	33.7
pO_2_ (mmHg)	62.5	120	84.6	97	104
Lactate (mmol/L)	6.8	4.6	2.4	1.5	1.8
APTT (s)	59.7	52.7	50.1	46.2	37.9
Serum creatinine (μmol/L)	530	367	—	183	141
Ventilatory parameters					
Respiratory rate	25	12	18	14	14
Oxygen saturation	90	100	97	98	100
Ventilation mode	VC	PC	PC	PC	PC
FiO_2_ (%)	100	35	35	35	45
IMV (times/min)	15	10	12	12	12
PEEP (cm H_2_O)	10	10	10	10	10
Tidal volume (mL)	550	470	486	455	578
ECMO record					
Sweep gas flow (SGF) L/min	—	4.0	4.0	0	—
ECMO flow (L/min)	—	4.1	4.0	3.2	—
FiO₂ ECMO (%)	—	100	60	21	—

On day 7 of VV-ECMO support, oxygenation improved significantly. The FiO₂ on ECMO was reduced to 21%, ventilatory support was minimized, and the PaO₂/FiO₂ ratio stabilized above 200, indicating recovery of autonomous oxygenation. Considering infection control, improved lung function, and stable hemodynamics, the team decided to wean the patient from VV-ECMO on day 8. Post-ECMO, the patient remained on mechanical ventilation with stable oxygenation and no major complications.

By day 9, follow-up chest CT showed significant improvement in lung conditions (Figure 2F). However, neck MRI (Figure 3A) and oropharyngeal CT (Figure 3B) indicated residual neck soft tissue swelling and glottic edema, though both had improved compared to prior findings. After 9 days of intubation and mechanical ventilation, the team considered extubation on day 10. MRI, CT, and laryngoscopy confirmed persistent but improving glottic edema (Figure 3C). Given the risk of infection associated with tracheostomy, extubation was attempted despite incomplete resolution of edema and a leak test indicating approximately 100 mL of leakage. However, within 1 h of extubation, oxygen saturation deteriorated, and the patient was reintubated.

On day 11, a tracheostomy was performed. The patient was transitioned to high-flow oxygen therapy and monitored closely for airway status. Due to neck soft tissue infection, the antibiotic regimen was adjusted to omadacycline (0.1 g IV daily, with an initial loading dose every 12 h on day 1). Postoperatively, the patient experienced chills, fever, and a rapid increase in PCT (>100 ng/mL). With the adjusted antimicrobial therapy, inflammatory markers rapidly improved, and no hemodynamic instability occurred.

**Figure 4:**
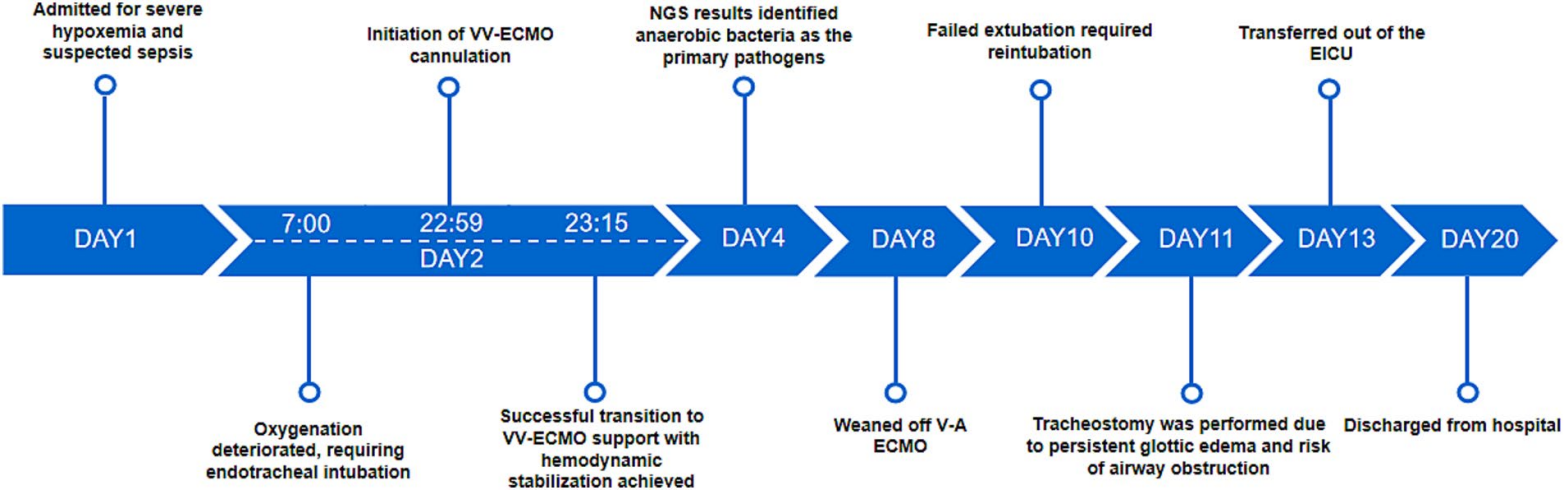
Patient’s treatment course.

The patient showed progressive recovery under continuous monitoring and treatment. On day 13, he was transferred to the general ward for further care. By day 20, he was discharged in good condition. Follow-up assessments indicated stable physical and mental health (Figure 4).

## Discussion

This case report describes a patient with mediastinal and cervical subcutaneous emphysema, sepsis, and severe ARDS caused by an anaerobic infection. Conventional treatments initially failed to improve the patient’s hypoxemia, highlighting the limitations of standard approaches in managing multi-organ damage caused by complex infections. By leveraging NGS for precise pathogen detection and guiding antimicrobial therapy, combined with VV-ECMO for oxygenation support, the patient’s condition significantly improved, and VV-ECMO was successfully weaned. This case underscores the potential of VV-ECMO in managing septic shock and severe ARDS, particularly in scenarios of rapid respiratory failure unresponsive to conventional treatments. Furthermore, the utility of NGS in the rapid identification of pathogens and tailoring individualized therapy provides critical support for the management of complex infectious cases. This successful outcome offers valuable insights into the combined use of VV-ECMO and NGS technology in infectious ARDS patients.

Anaerobic bacteria utilize unique metabolic pathways to break down organic matter in hypoxic environments, producing gases such as hydrogen and carbon dioxide. These gases can spread through anatomical planes (e.g., the retropharyngeal space and deep fascial layers), resulting in mediastinal and subcutaneous emphysema (4). Anaerobic bacteria also secrete hyaluronidase and collagenase, which degrade host tissue barriers, accelerating gas dissemination and infection spread (1). Additionally, The rapid progression of anaerobic infections is closely associated with tissue necrosis and reduced redox potential, which creates an ideal environment for anaerobic bacterial proliferation and gas production. In this case, imaging studies revealed extensive mediastinal and cervical subcutaneous emphysema. NGS enabled the rapid identification of pathogens, demonstrating its advantages as a high-throughput diagnostic tool. In addition to identifying complex polymicrobial infections, NGS can detect anaerobic bacteria that are challenging to identify with conventional culture methods. Compared to traditional culture, NGS offers significant advantages in speed and sensitivity (5).

VV-ECMO is commonly used to treat refractory hypoxemia and severe ARDS (3), but its application in septic shock remains controversial (6). Septic shock patients are often deemed unsuitable for ECMO support due to hemodynamic instability and systemic inflammatory responses (7). However, this case demonstrates the successful use of VV-ECMO in a unique clinical scenario. When conventional mechanical ventilation and antimicrobial therapy failed, and the patient developed severe hypoxemia and cardiopulmonary arrest, the timely initiation of VV-ECMO significantly improved oxygenation and provided critical time for infection control and organ recovery. Regarding ECMO mode selection, bedside echocardiography excluded significant cardiac dysfunction, confirming that the patient’s ARDS was primarily due to lung injury, thus favoring VV-ECMO over VA-ECMO. Studies suggest that VV-ECMO provides targeted oxygenation support in respiratory failure while avoiding the additional hemodynamic burden associated with VA-ECMO (8, 9). Furthermore, thorough pre-ECMO assessments, including chest CT, echocardiography, and hemodynamic monitoring, were essential for guiding treatment decisions. Although VV-ECMO was effective in this case, the potential risks associated with its use in septic shock, such as infection dissemination, thrombosis, and complications, warrant attention. Literature suggests that dynamic monitoring of infection markers and optimizing antimicrobial regimens can enhance the safety of VV-ECMO in ARDS induced by sepsis (9).

Airway management is a critical aspect of treating patients with glottic edema and cervical soft tissue infections, requiring careful consideration of multiple factors in decisions regarding tracheostomy and extubation (10). Glottic edema is a leading cause of extubation failure, and extubation should typically be preceded by laryngoscopic examination, leak tests, and respiratory parameter monitoring (11, 12). Studies indicate that attempting extubation is reasonable when glottic edema has partially resolved, but premature extubation in cases of significant edema or failed leak tests may increase the risk of extubation failure (13). In this case, the patient experienced oxygenation deterioration after extubation due to glottic edema and cervical infection, necessitating reintubation and eventual tracheostomy. Despite the risk of soft tissue infection increasing bacteremia, preoperative infection control, close monitoring of PCT and CRP, and timely adjustments to antimicrobial therapy enabled successful management of postoperative infections. This experience demonstrates that tracheostomy can be a reasonable option even in high-risk situations, provided it is guided by dynamic assessments and precise treatment. Delaying extubation allows further resolution of glottic edema but increases the risk of pulmonary infections due to prolonged intubation. Early tracheostomy, while reducing extubation failure, carries a higher risk of infection. In this case, the combination of laryngoscopic evaluation and infection control measures ultimately supported tracheostomy as the airway management solution, offering valuable insights for similar scenarios.

This case report highlights the unique application of VV-ECMO in septic shock and severe ARDS, along with airway management strategies. However, there are several limitations. First, as a single case report, this study lacks control groups and long-term follow-up data, preventing a systematic evaluation of the generalizability and long-term outcomes of VV-ECMO. Second, the diagnosis relied primarily on NGS for pathogen detection without direct comparison to traditional culture methods, limiting the validation of NGS sensitivity and specificity. Finally, multiple interventions, including VV-ECMO, antimicrobial therapy, and tracheostomy, were employed in this case, and the independent contributions of each intervention to the outcome remain unclear. Future research, including multicenter studies, large sample sizes, and randomized controlled trials, is needed to further validate the safety and efficacy of VV-ECMO combined with comprehensive therapeutic strategies in similar cases.

## Conclusion

This case report demonstrates the successful application of VV-ECMO in managing septic shock complicated by severe ARDS, combined with NGS technology for precise pathogen detection and individualized antimicrobial therapy. The airway management strategies for glottic edema and soft tissue infections provide valuable references for similar cases. Although the use of VV-ECMO in the context of septic shock remains controversial, this case highlights its potential value in specific clinical scenarios. Further research is warranted to validate its applicability and efficacy.

## Data Availability

The original contributions presented in the study are included in the article/supplementary material, further inquiries can be directed to the corresponding author.
